# Comparison of polyvinyl chloride and elastic stannum stylet extraction forces with various lubricants: a simulation study

**DOI:** 10.1186/s40064-016-2985-8

**Published:** 2016-08-09

**Authors:** Takanobu Fujisawa, Nobuyasu Komasawa, Haruki Kido, Toshiaki Minami

**Affiliations:** Department of Anesthesiology, Osaka Medical College, Daigaku-machi 2-7, Takatsuki, Osaka 569-8686 Japan

**Keywords:** Polyvinyl chloride stylet, Stannum stylet, Lubricant, Extraction force, Simulation

## Abstract

**Background:**

Stylets are the most frequently used devices for tracheal intubation, but can be a source of postoperative pharyngeal pain or hoarseness. In this study, we evaluated extraction forces between polyvinyl chloride (PVC) and stannum (Sn) stylets with various lubricants.

**Findings:**

Using a manikin, we compared extraction forces between PVC and Sn stylets under four different conditions: without lubricant, 3 ml of water (water), three sprays of 8 % lidocaine (lidocaine), and olive oil. A force measuring device was used to accurately measure the extraction force for stylet removal. The extraction force was significantly smaller with the Sn stylet compared to the PVC stylet, regardless of the lubricant used for all three tracheal tubes with different diameters tested (*P* < 0.05). In comparisons by lubricant, lidocaine and olive oil resulted in significantly lower extraction forces than with no lubricant or water with the PVC stylet for all tracheal tubes tested. In contrast, there were no significant differences in extraction force by lubricant for the Sn stylet across all tracheal tubes tested.

**Conclusions:**

The Sn stylet required less extraction force compared to the PVC stylet, regardless of the lubricant used.

## Background

Although several types of devices for tracheal intubation exist, such as videolaryngoscopes (Asai et al. 2009), the most widely used intubation supporting device is the stylet. Stylets render the tracheal tube more rigid and make it easier to insert them into the trachea. However, excessive force or rough maneuvers can damage the pharyngeal and tracheal anatomy, leading to pharyngeal pain or prolonged hoarseness. In this context, stylet extubation force may not be negligible, particularly since the rigid stylet is extracted from a curved tracheal tube.

Stylets made of stannum (Sn) have been recently developed and commercially available in Japan. These stylets are deformable and lubricious. Anesthesiologists can shape the stylet manually with no problem. We hypothesized that they would require less extraction force compared to conventional stylets. As clinical comparisons would likely present ethical issues, we conducted a simulation study to evaluate the extraction force required for conventional polyvinyl chloride (PVC) and Sn stylets when used in combination with different types of lubricants.

## Methods

**Fig. 1:**
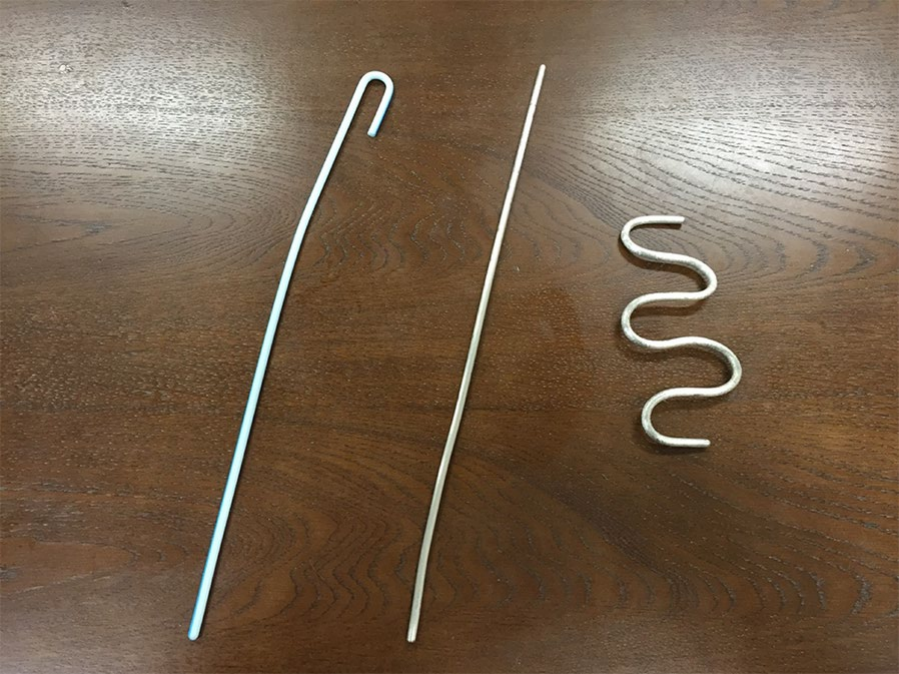
Images of the PVC stylet and Sn stylet. PVC stylet (*left*), Sn stylet (*middle*), and Sn stylet with curvature (*right*)

The Airway Trainer^®^ manikin (Laerdal, Sentrum, Stavanger, Norway) was used for intubation and stabilization of the tracheal tube. Tracheal tubes (Portex Soft Seal^®^, Smith Medical Co Ltd., Minnesota, USA) with different internal diameters were tested. A PVC stylet (Tracheal Intubation Stylet^®^, Smith Medical Co Ltd., Minnesota, USA) with an external diameter of 5 mm, or a stannum (Sn) stylet (elastic stylet, Total Medical Company, Toyama, Japan) with the same diameter, was inserted into the tracheal tube, which was then placed into the trachea at a depth of 23 cm from the incisors using the Macintosh laryngoscope (Fig. 1). The tracheal tubes were fixed firmly with a tube holder (Portex Soft Seal^®^, Smith Medical Co Ltd., Minnesota, USA) (Komasawa et al. 2015).

To assess the effects of lubricants on stylet extraction force, we compared four different conditions: without lubricant, 3 ml of water (water), three sprays of 8 % lidocaine (lidocaine), and olive oil. A force measuring device (Digital Force Gauge^®^, Shimpo Co Ltd., Tokyo, Japan) was used to accurately measure stylet extraction force, which contains accuracy down to 1 decimal places of newton according to the manufacturer (Mihara et al. 2015). Force measurements are expressed in Newtons. All trials were conducted by the same anesthesiologist to unify conditions. Measurements were performed 5 times for each condition.

Results obtained from each trial were compared by two-way repeated measures analysis of variance and Tukey’s multiple comparison test were used for each comparison. Data are presented as mean ± SD. *P* < 0.05 was considered statistically significant.

## Results

**Fig. 2:**
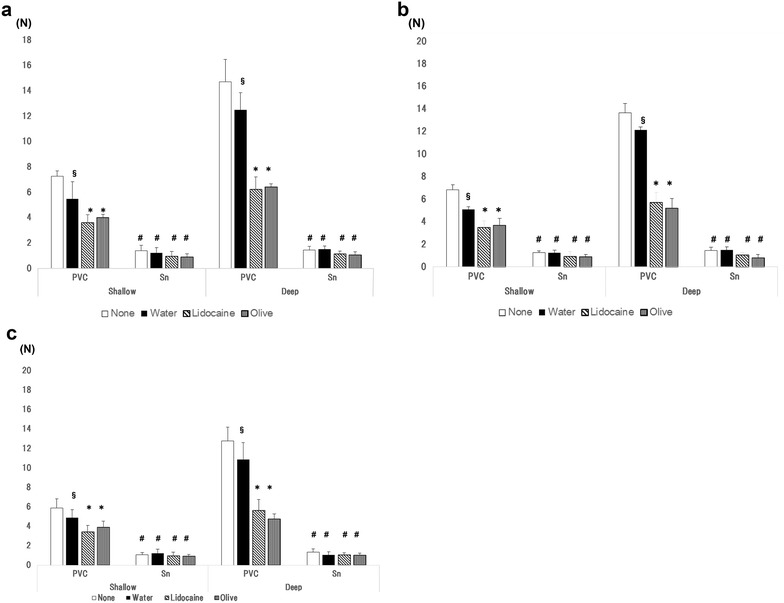
Comparison of extraction forces with different lubricants. **a** Tracheal tube with an internal diameter (ID) of 7.0 mm, **b** tracheal tube with an ID of 7.5 mm, **c** tracheal tube with an ID of 8.0 mm. None: stylets were not treated with any lubricant; Water: stylet pre-treated with water; lidocaine: stylet pretreated with lidocaine spray; olive: stylet pretreated with olive oil. ^#^ *P* < 0.05 compared to PVC stylet. *P < 0.05 compared to none or water. ^§^ *P* < 0.05 compared to none

Extraction forces for the various conditions are shown in Fig. 2. In the shallow trial, the extraction force was significantly smaller with the Sn stylet than with the PVC stylet, regardless of the lubricant used for all three tracheal tubes of different internal diameters (I.D. 7.0 mm; None 7.3 ± 0.4 N, Water 5.5 ± 1.3 N, Lidocaine 3.6 ± 0.6 N, Olive 4.0 ± 0.6 N in PVC trial, and None 1.4 ± 0.4 N, Water 1.2 ± 0.4 N, Lidocaine 0.9 ± 0.4 N, Olive 0.9 ± 0.2 N in Sn trial, I.D. 7.5 mm; None 6.8 ± 0.4 N, Water 5.1 ± 1.0 N, Lidocaine 3.5 ± 0.6 N, Olive 3.7 ± 0.6 N in PVC trial, and None 1.2 ± 0.2 N, Water 1.2 ± 0.4 N, Lidocaine 0.9 ± 0.4 N, Olive 0.9 ± 0.2 N in Sn trial, I.D. 8.0 mm; None 5.9 ± 0.6 N, Water 4.9 ± 0.8 N, Lidocaine 3.4 ± 0.7 N, Olive 3.9 ± 0.6 N in PVC trial, and None 1.1 ± 0.2 N, Water 1.2 ± 0.4 N, Lidocaine 0.9 ± 0.4 N, Olive 0.9 ± 0.2 N in Sn trial; each *P* < 0.05).

In the deep trial the extraction force also showed with the Sn stylet than with the PVC stylet, regardless of the lubricant used for all three tracheal tubes (I.D. 7.0 mm; None 14.7 ± 1.84 N, Water 12.5 ± 1.3 N, Lidocaine 6.2 ± 1.0 N, Olive 6.4 ± 1.6 N in PVC trial, and None 1.4 ± 0.3 N, Water 1.5 ± 0.3 N, Lidocaine 1.1 ± 0.2 N, Olive 1.1 ± 0.2 N in Sn trial, I.D. 7.5 mm; None 13.6 ± 0.8 N, Water 12.1 ± 1.5 N, Lidocaine 5.7 ± 0.9 N, Olive 5.2 ± 0.8 N in PVC trial, and None 1.4 ± 0.3 N, Water 1.5 ± 0.3 N, Lidocaine 1.1 ± 0.2 N, Olive 0.8 ± 0.3 N in Sn trial, I.D. 8.0 mm; None 12.8 ± 1.4 N, Water 10.9 ± 1.8 N, Lidocaine 5.6 ± 1.1 N, Olive 4.7 ± 0.5 N in PVC trial, and None 1.3 ± 0.3 N, Water 1.1 ± 0.3 N, Lidocaine 1.0 ± 0.2 N, Olive 1.0 ± 0.2 N in Sn trial; each *P* < 0.05).

In comparisons by lubricant, the extraction force of the PVC stylet was significantly smaller with lidocaine and olive oil compared to with no lubricant or water for all tracheal tubes tested. In contrast, no significant differences were observed in extraction force across all lubricants for the Sn stylet.

## Discussion

We previously showed that stylet extraction force correlates with postoperative pharyngeal pain and extraction force of more than 10.3 N was obtained as a cutoff level for postoperative sore throat (Kusunoki et al. 2016). Thus, we focused on differences in stylet extraction force by lubricants and stylets in this study. In this study, the extraction force was over 10.3 N in PVC trials with none lubricant or water, suggesting the risk of pharyngeal pain in clinical settings.

In this study, lidocaine and olive oil reduced the stylet extraction force for the conventional PVC stylet. The Sn stylet required less extraction force than the PVC stylet, regardless of the lubricant used, likely due to its deformable and lubricious nature. These findings confirm the utility of the Sn stylet for tracheal intubation.

This study has several limitations. First, we used a manikin rather than real patients. The softness and anatomical structure differ between manikins and real patients. Second, we evaluated only one type of tracheal tube (although we tested different internal diameters) for stylet extraction. Evaluation of other tracheal tube types is warranted in the future (Komasawa et al. 2014). Third, we did not include silicone as lubricants (Taylor et al. 2012). Fourth, there is a possibility that the external diameter of PVC or Sn stylet were not strictly similar.

In the future, randomized clinical trials comparing the efficacy and complications on Sn and PVC stylets are warranted.

In conclusion, our simulation study demonstrated that the Sn stylet requires less extraction force compared to the PVC stylet, regardless of the lubricant used.
